# Comparative Assessment of Physiological Responses to Emotional Elicitation by Auditory and Visual Stimuli

**DOI:** 10.1109/JTEHM.2023.3324249

**Published:** 2023-10-12

**Authors:** Edoardo M. Polo, Andrea Farabbi, Maximiliano Mollura, Alessia Paglialonga, Luca Mainardi, Riccardo Barbieri

**Affiliations:** DEIBPolitecnico di Milano 18981 20133 Milan Italy; DIAGSapienza University of Rome 9311 00185 Rome Italy; Cnr-Istituto di Elettronica e di Ingegneria dell’Informazione e delle Telecomunicazioni (CNR-IEIIT) 20133 Milan Italy

**Keywords:** Biomedical signal processing, emotion elicitation, international affective digital sounds (IADS), international affective pictures system (IAPS), physiological responses

## Abstract

The study of emotions through the analysis of the induced physiological responses gained increasing interest in the past decades. Emotion-related studies usually employ films or video clips, but these stimuli do not give the possibility to properly separate and assess the emotional content provided by sight or hearing in terms of physiological responses. In this study we have devised an experimental protocol to elicit emotions by using, separately and jointly, pictures and sounds from the widely used International Affective Pictures System and International Affective Digital Sounds databases. We processed galvanic skin response, electrocardiogram, blood volume pulse, pupillary signal and electroencephalogram from 21 subjects to extract both autonomic and central nervous system indices to assess physiological responses in relation to three types of stimulation: auditory, visual, and auditory/visual. Results show a higher galvanic skin response to sounds compared to images. Electrocardiogram and blood volume pulse show different trends between auditory and visual stimuli. The electroencephalographic signal reveals a greater attention paid by the subjects when listening to sounds compared to watching images. In conclusion, these results suggest that emotional responses increase during auditory stimulation at both central and peripheral levels, demonstrating the importance of sounds for emotion recognition experiments and also opening the possibility toward the extension of auditory stimuli in other fields of psychophysiology. Clinical and Translational Impact Statement- These findings corroborate auditory stimuli’s importance in eliciting emotions, supporting their use in studying affective responses, e.g., mood disorder diagnosis, human-machine interaction, and emotional perception in pathology.

## I. Introduction

Emotions are defined as a physiological mental state which involves people’s reactions after the onset of internal or external stimuli. As such, they are strongly affected by subjective experience [1]. Emotions are fundamental in our life since they make us survive and reproduce through adaptation to the environment [2]. They are generated by a very complex aggregate of neuronal and hormonal interactions which strongly influence decision-making processes [3]. Emotion-related studies are gathering much interest in our society since they can be extended in many fields such as the new horizons of Human-Computer and Human-Robot interaction [4] but also in more clinical applications as the monitoring of depression states, anxiety, stress, chronic anger and mood disorders. Thus, devising methodologies to properly elicit and recognize emotions can largely help in assessing and quantifying diseases which influence human health and wellbeing.

A great interest is rising on the study of physiological signals and how they change in response to different elicited emotions. This interest is mainly due to the fact that physiological signals are linked above all to the autonomic nervous system (ANS) and can be easily acquired by wearable devices [5]. Moreover, physiological reactions are objective in nature and are not influenced by subjective judgment.

In the literature there are many models which classify emotions and can be divided in two principal categories: categorical and dimensional. In the first case emotions are described with a limited number of innate and universal categories, easily distinguishable between each other (e.g. anger, fear, joy, surprise, sadness) [6] while in the second case each emotion can be explained as the linear combination of specific and independent dimensions. The most used dimensional model in literature is the Russel’s Circumplex model [7] which identifies two principal dimensions: *arousal*, which represents the level of excitation that an emotion can elicit and *valence*, which is the level of pleasantness/unpleasantness carried by the emotion itself. Difficulties in describing emotions as categorical have led several researchers to make use of dimensional models since emotions are not discrete entities but are more blurred experiences which often overlap with each other [8].

Most studies in the field of affective computing use pictures as stimuli, predominantly the International Affective Pictures System (IAPS) [9]. These pictures are widely used for experiments about emotions as they possess valence and arousal values obtained over the years as an average given by the labeling of thousands of people and for this reason are well suited stimuli for emotion recognition, for example using computational models. Very few studies use instead auditory stimuli from the International Affective Digital Sounds (IADS) [10]. The IADS is homologue to the IAPS database, but it contains standardized sounds in valence and arousal levels.

Although a soundtrack in video-clips is a part of the stimulus itself, it is not easy to understand what kind of stimulation such as audio, visual or audio-visual is more effective in eliciting emotions. To the best of our knowledge, almost no study used the combination of audio and video stimuli [11]. Moreover, there are more recent studies in the literature which created emotion databases by acquiring physiological signals and using films or video-clips as stimuli [12], [13]. From the creation of these databases, other studies arised with the purpose of achieving highest possible levels of accuracy in discriminating the different emotions elicited through machine learning models [14], [15]. However, in most of these studies little importance is given to the interpretation of the features extracted from the signals and, also, limited evidence is provided to better understand which signals are most effective to assess the relation between emotions and physiology.

In [11], which is one of the very few studies using both IAPS and IADS separately and together, authors found a general heart rate (HR) deceleration in response to emotional stimulation. The observed HR deceleration was independent from the stimulation modality and more emphasized in response to negative stimuli as compared with responses to positive and neutral stimuli. In [16], which uses IAPS and IADS separately, results showed that heart rate responses are unique for every category of the emotional stimuli with an higher rate of heart rate deceleration when listening to sounds only. In our research, we have introduced an innovative approach to elicit emotions. We utilized the well-established IAPS and IADS datasets, both individually and in combination, while simultaneously monitoring various physiological signals. This study’s novelty lies in its use of these validated datasets to compare different emotional stimulation methods. While most studies primarily focus on visual stimuli, incorporating sound is rare, and even rarer is the use of both modalities with these datasets. Furthermore, in this study we tracked five different physiological signals. Our objective is to examine the physiological patterns associated with various stimulation methods and evaluate their effectiveness. This study places particular emphasis on analyzing responses triggered by auditory stimuli to quantify their influence on emotional responses and to explore their potential in diverse psycho-physiological contexts. Preliminary findings of this research were presented in [17] and [18].

## II. Methods and Procedures

### A. Study Design and Data

Our experimental protocol includes 13 female and 8 male (age: 26.18 ± 1.47 years) volunteers. The experiments were performed at SpinLab of Politecnico of Milano after all subjects signed an inform consent approved by the Politecnico di Milano Reasearch Ethical Committee (Opinion n. 29/2021). During the experiment, the electrocardiogram (ECG), blood volume pulse (BVP), galvanic skin response (GSR), pupillary signal (PUPIL) and electroencephalogram (EEG) were acquired. ECG, BVP and GSR were acquired by Procomp Infiniti device with a sampling frequency fixed at 256 for GSR and 2048 Hz for ECG and BVP. PUPIL was acquired by Tobii Pro X2 Compact eye-tracker with a sampling frequency of 60 Hz. The EEG signal was acquired with the DSI 24 headset composed by 21 dry electrodes located at: Fp1, Fp2, Fz, F3, F4, F7, F8, Cz, C3, C4, T7/T3, T8/T4, Pz, P3, P4, P7/T5, P8/T6, O1, O2, A1, A2. The electrodes location follows the international 10–20 system. The headset acquired data with a sampling rate of 300 Hz with and A/D converter at 16 bits. Participants were seated on a comfortable chair during the experiment. To minimize behavioral heterogeneity, participants were asked to avoid coffee and smoke for two hours prior to the experiment. During experiments one experimenter was always present in the room to manage possible problems with the acquisitions and prevent any pronounced movement of the subjects or detachments of sensors. All subjects were tested with the pure tone audiometry examination using a clinical audiometer (Amplaid 177+, Amplifon with TDH49 headphones) to ensure that their hearing thresholds were in the normal hearing range (pure tone average thresholds at 0.5, 1, 2, and 4 kHz < 20 dB HL). The acoustic stimulation was performed by using headphones (UXD CT887) and subjects with vision problems were able to use glasses. All participants were asked if they had any pre-existing medical conditions and/or mental disorders, and if so, they were excluded from the study. Regarding visual aspects, especially the pupillary signal, we adhered to established guidelines [19]. To reduce external light interference, we darkened the lab windows and used consistent artificial lighting during recording. Screen brightness remained constant for all participants. For the auditory component, participants were exposed to a sound before the test and were guided to choose a comfortable volume level.

The protocol consists of three randomized phases interspersed with 2 minutes rest:

1) IAPS-only
2) IADS-only
3) IAPS + IADS

This protocol extends an existing protocol in which IAPS and IADS have been used as stimuli separately on different subjects [20], [21]. Each of the above phase includes four sessions: after an initial resting period of 5 minutes while subjects look at a grey screen, four sessions at increasing arousal levels are alternated with neutral sessions of 90 seconds each.

Every arousing session includes six visual and/or acoustic stimuli, lasting 15 seconds each, having the first half with low valence and the second half with high valence. The experiment lasts 47 minutes. The rationale is to slowly increase the arousal maintaining a median neutral valence in each session. Neutral sessions deliver low arousal stimuli with neutral valence which serve as a baseline interlude of rest between two emotional sessions.

**FIGURE 1.**
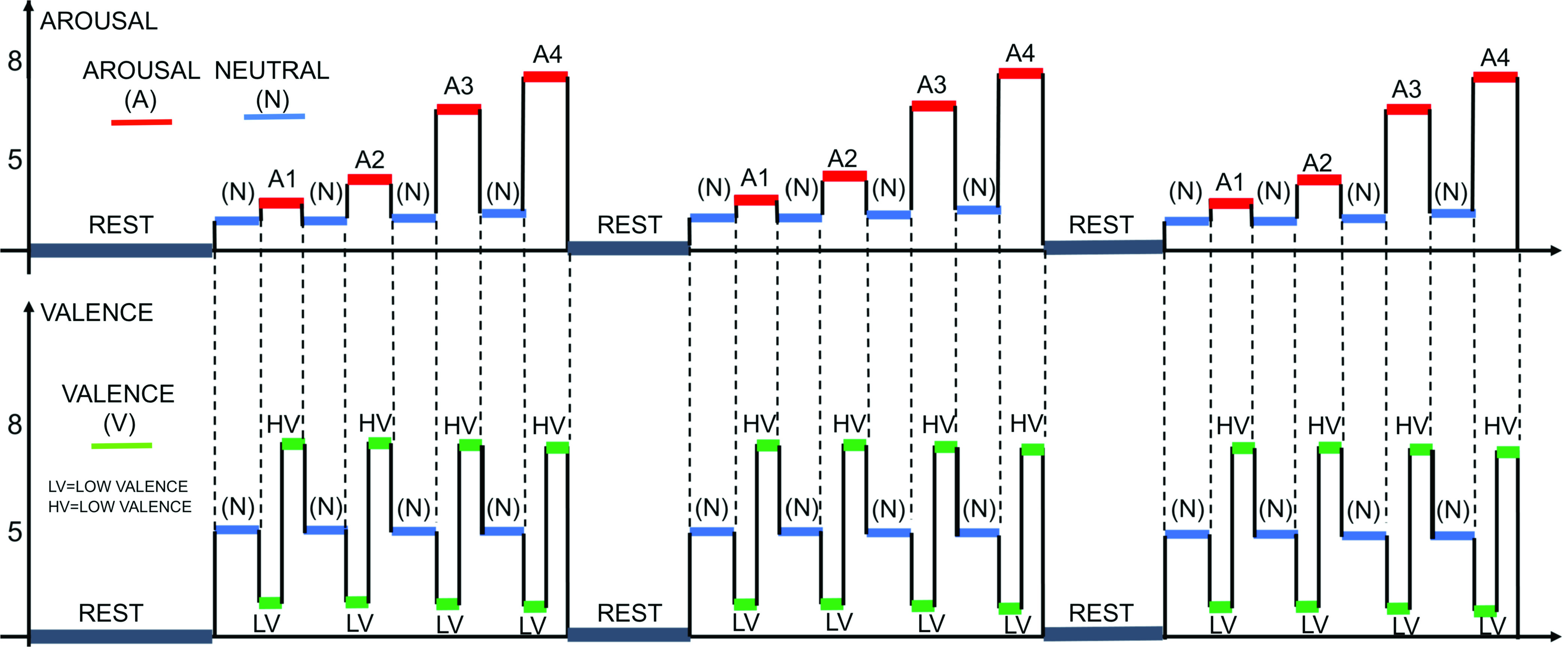
Outline of the experimental protocol, including three stimulation phases. Top panel: arousal (red). Bottom panel: valence (green).

In IAPS+IADS, pictures are delivered with simultaneous sounds chosen to have a semantic match (e.g. picture of a dog growling and the sound of a dog barking). In IAPS+IADS, pictures and sounds belonging to the neutral sessions of the IAPS-only and IADS-only are delivered together. Figure 1 shows the experimental protocol. Arousal and valence levels are set according to IAPS and IADS scores as reported in Table 1. In order to compare IAPS and IADS labeling with the subjective one, immediately after the end of the protocol, participants were asked to perform a self-assessment of the stimuli seen and heard during the experiment. Each stimulus was quickly reproduced and subjects had to select arousal and valence levels using self-assessment manikins according to their own emotional reaction. Self-assessment manikins are a pictorial and non-verbal questionnaire to evaluate emotions [22] in which low valence is represented by a sad manikin increasingly smiling moving towards higher valence levels while arousal is painted used manikins showing relaxation for lower levels and excitement for higher levels. The average subjective ratings of values and arousal were aligned with the valence and arousal values shown in Table 1, suggesting that the stimuli here used were effective on both negative and positive valence dimension and on the increasing arousal dimension.

**TABLE 1:** Valence and Arousal Medians and Ranges for all Sessions (Neutral, Arousal1, Arousal2, Arousal3 and Arousal4) of Each Phase (IAPS-only, IADS-Only and IAPS+IADS, Which Comprises Matched IAPS and Matched IADS

Session	Valence Rating	Arousal Rating
IAPS only		
Neutral	5.02(4.39–5.55)	3.36(3.07–3.84)
Arousal1	4.96(3.92–7.24)	4.68(4.42–4.85)
Arousal2	4.81(2.52–7.62)	5.38(5.08–5.48)
Arousal3	5.10(2.14–7.2)	6.55(6.09–6.80)
Arousal4	4.75(1.45–7.57)	7.12(6.90–7.35)
IADS only		
Neutral	5.47(4.34–5.99)	3.74(2.88–4.15)
Arousal1	5.16(3.54–7.12)	4.77(4.47–4.94)
Arousal2	4.93(2.46–7.78)	5.40(5.05–5.87)
Arousal3	4.88(2.44–7.38)	6.35(6–6.84)
Arousal4	4.86(1.68–7.67)	7.14(7.03–7.88)
matched IAPS		
Arousal1	5.67(3.65–7.13)	4.46(4.13–4.75)
Arousal2	4.44(2.49–6.83)	5.43(5.18–5.53)
Arousal3	4.76(2.16–6.83)	6.21(6.06–6.79)
Arousal4	4.19(1.48–7.61)	7.18(7.13–7.31)
matched IADS		
Arousal1	5.86(4.52–7.05)	4.62(4.38–4.87)
Arousal2	4.82(3.02–6.11)	5.50(5.34–5.74)
Arousal3	4.54(2.06–6.77)	6.42(6.07–6.64)
Arousal4	4.54(1.99–6.94)	7.35(7.28–8.16)

### B. Data Processing and Feature Extraction

In the following subsections the processing and the feature extraction steps are explained for each signal.

#### 1) GSR

GSR was filtered at 2 Hz with a zero-phase low pass Butterworth of 4th order and it was then downsampled at 5 Hz. In order to separate the phasic component from the entire signal, a median filter was applied. Specifically, each sample amplitude was replaced by the median amplitude of the surrounding samples in a time window of +/−4 seconds centered on the current sample [23]. The resulting median signal was then subtracted from the filtered one and the resulting phasic signal was used to extract GSR peaks linked to the eccrine glands’ spikes. GSR peaks were computed as local maxima on the filtered signal between each onset (amplitude $ > 0.01~\mu \text{S}$) and offset ( $0~\mu \text{S} < $ amplitude) occurrences on the phasic signal. Given the relative slowness of the signal and having to consider short time windows to be analyzed, we focused only on time features. In particular we extracted:

• the number of GSR peaks (*GSR n peaks*)
• the amplitude of GSR peaks (*GSR Amp peaks*)
• the average rise and recovery time of GSR peaks (*AVGSR rise time*, *AVGSR recovery time*)
• GSR average (*AVGSR*) and standard deviation (s.d.) (*SDGSR*)
• the maximum signed amplitude between two consecutive maxima (*MAXGSR sign amp*) computed on the signal filtered with a Butterworth band-pass filter of the 2th order from 0.5 to 1 Hz
• the average (*AVGSR der*), s.d. (*SDGSR der*) and maximum of the first derivative (*MAXGSR der*) of the signal
• the envelope of the phasic component computed as the average of its integral *(AVenv*)

#### 2) ECG

A 4th order zero-phase low-pass Butterworth anti-aliasing filter with a cut-off frequency at 125 Hz was applied to the ECG signal in order to remove high frequency noise coming both from the acquisition system and eventual subjects’ movements. Subsequently, the signal was downsampled at 250 Hz and R-peak locations were extracted by means of a Pan-Tompkins based algorithm [24]. All R-peaks locations were then checked and any mistake in the annotation was manually corrected through a in-house software. In order to deal with non-stationarities of physiological responses and with the intrinsic non-stationary nature of the emotion stimuli as well as the short duration of the stimuli, we applied a Point Process framework to model the inter-beat interval (RR) series. Given the intrinsic Point Process nature of the RR intervals relying on the R-wave events this model well fits as a framework for modeling the heartbeat [25]. More specifically, the inter-beat-interval series was modelled according to the following history-dependent inverse gaussian distribution:

\begin{equation*} p(t)=\left ({{\frac {\theta _{p+1}}{2\pi (t-R_{k})^{3}}} }\right)^{\frac {1}{2}} \exp \left ({-\frac {\theta _{p+1}(t-R_{k}-\mu _{RR})}{2~\mu _{RR}^{2}(t-R_{k})}}\right) \tag{1}\end{equation*}

whose expected value is estimated with an autoregressive (AR) model as follows:

\begin{equation*} \mu _{RR}= \theta _{0} + \sum _{i=1}^{p} \theta _{i}(t) RR_{k-i} \tag{2}\end{equation*}

where $RR_{k}$ represents the $k$-th RR interval closer to time $t$, and $\theta _{0:p}$ and $\theta _{p+1}$ parameters represent the AR model parameters and the shape parameter of the inverse gaussian, which are continuously estimated through local maximum likelihood estimation according to [25]. AR coefficients were then used to compute a time-varying spectral analyses of the RR interval series. In this way we were able to track very fast stimulus-response changes. From the resulting time-varying estimation we were able to extract averaged features in each emotion time window such as:

• the modelled RR series ( $\mu $ *RR*) and the variability of the inverse gaussian distribution ( $\sigma 2$)
• the RR power spectral density of the modelled RR series in very low (*RR VLF*) [< 0.04 Hz], low (*RR LF*) [0.04-0.15 Hz] and high (*RR HF*) [0.15-0.5 Hz] frequency ranges and the sympatho-vagal balance index (*RR LF/HF*)
• the normalized power spectral density of the modelled RR series in low (*RR LFn*) and high (*RR HFn*) frequency ranges and the total power spectral density of the modelled RR series *(RR TOT*)

**FIGURE 2.**
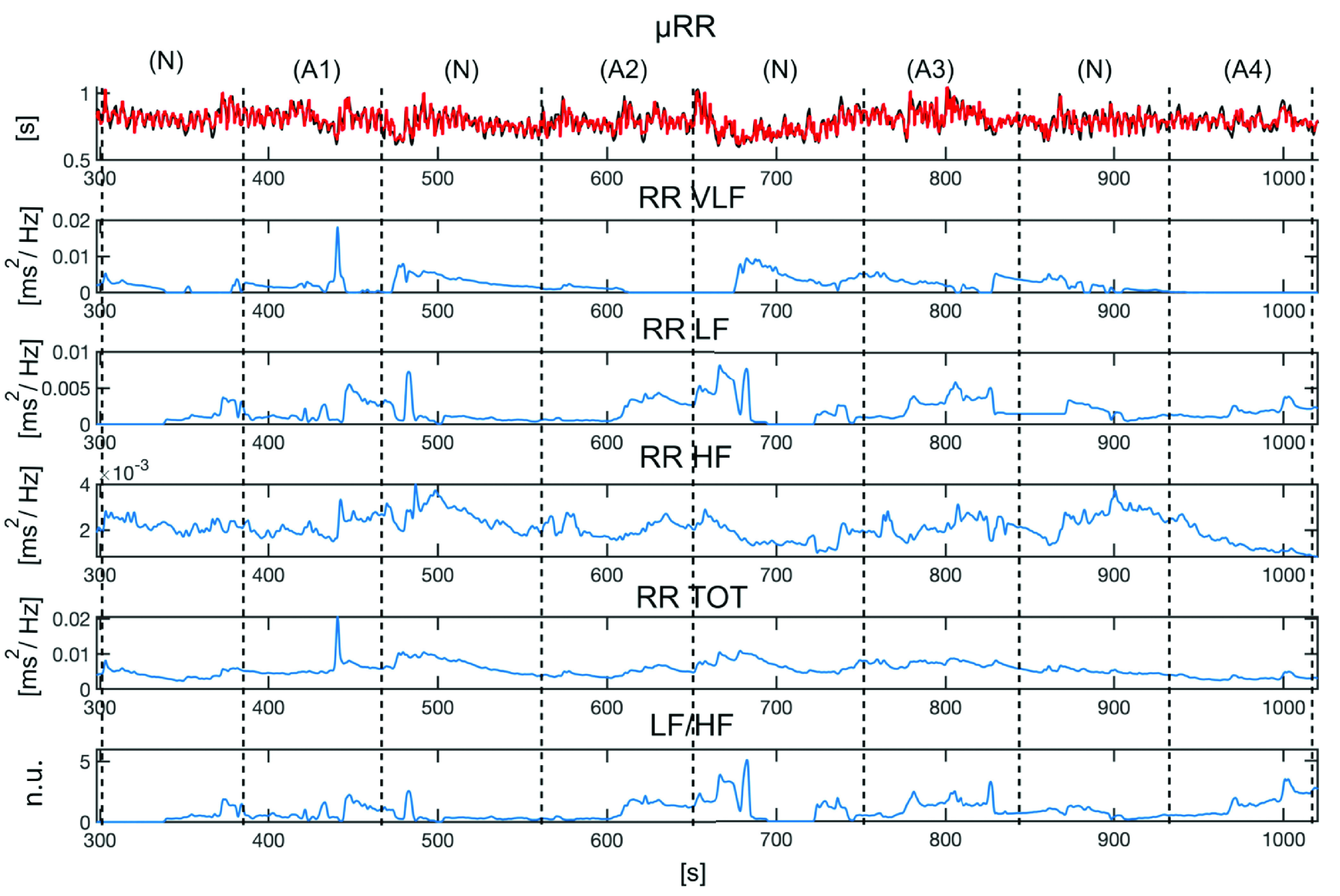
Instantaneous HRV tracking resulting from the point process modeling for ‘subject1’. In the highest panel both the RR series and the modelled RR series ( $\mu $RR) are shown in black and red, respectively. In the subsequent panels, the principal HRV indices are shown.

Figure 2 shows for the first phase (720 seconds) of ‘subject1’ the instantaneous tracking of the heart rate variability (HRV) indices extracted.

#### 3) BVP

BVP was filtered by a 4th order zero-phase low-pass Butterworth anti-aliasing filter with a cut-off frequency at 25 Hz and then it was downsampled at 250 Hz. Locations and related amplitude values of systolic, diastolic and onset fiducial points were extracted starting from the R-peaks annotation on the ECG signal. More specifically, the maximum and minimum BVP values between two consecutive R-peaks were considered as systoles and diastoles, respectively. Onset values were found by looking at each inflection point between each systolic and diastolic location. All locations and relative amplitudes of the three fiducials points were checked to be sure of the their correctness and eventual mistakes were manually corrected. As for the ECG signal, the signal processing was done considering the entire signal, then fiducial points’ locations and feature extraction took place in shorter windows. From BVP and its link with ECG, two features were extracted in the emotion time windows:

• The average amplitude difference between each systolic and the corresponding diastolic value, computed and referred as a index of the average volume pulse (*VP*)
• The average pulse arrival time (*PAT*), computed as the average time difference between each onset value on the BVP signal and the corresponding R-peak on the ECG

For the PAT computation onsets were used with respect to diastoles or systoles since they reveal to be more stable points given the lower indecision on the correct position in the noisiest signal sections.

#### 4) EEG

The EEG signal was processed in order to extract the relevant features. As the signal acquired by the electrode Pz was found to be corrupted, it was replaced by an interpolation obtained by the signals recorded at the nearest electrodes.

All signals were re-referenced through Common-Average Referencing (CAR) in order to remove common noise and filtered in the main brain waves oscillation frequencies as defined in [26]: $\delta (1-3Hz), \theta (4-7Hz), \alpha (8-12Hz), \beta (16-38Hz)$. It was decided to not include the $\gamma $ waves $(>38Hz)$ since literature suggests that no emotion-related brain activity happens at this frequencies and also to remove muscular artifacts manifesting at higher frequencies.

Regarding EEG, the following features were extracted in each emotion session:

• the power spectral density in the frontal and parietal regions for each frequency band ( $\delta (1-3Hz)$ *F*, $\delta (1-3Hz)$ *P*, $\theta (4-7Hz)$ *F*, $\theta (4-7Hz)$ *P*, $\alpha (8-12Hz)$ *F*, $\alpha (8-12Hz)$ *F*, $\beta (16-38Hz)$ *F*, $\beta (16-38Hz)$ *P*)
• the ratio of the power spectral densities $\beta /\theta $ in the frontal and parietal regions ( $\beta $ */* $\theta $ *F*, $\beta $ */* $\theta $ *P*) in order to assess the attention level of the subjects during the trials.

All power spectral densities were computed using the Welch-method. Concerning attention related features, the ratio $\beta /\theta $ is supposed to increase during attentive states [27].

#### 5) Pupil

All samples were checked and values lower than 2 mm or higher than 8 mm were replaced by NaN and treated as blinks since considered out of the physiological range [2-8 mm]. At first, for each time instant if only one eye had a blink then the sample value of the other eye was replaced to the missing sample. Secondly, where both eyes had blinks, a linear interpolation of the signal was carried out. Once all samples were within the physiological range, the signal was filtered by a 4th order zero-phase low-pass Butterworth anti-aliasing filter with a cut-off frequency at 5 Hz and then it was downsampled at 10 Hz.

The pupillary diameter was then computed as the average of the amplitude of both eyes. Both time and frequency features were then extracted on the processed signal in the emotion time windows. In particular, from PUPIL we extracted:

• the average of the diameter (*AVD*) and its s.d. (*SDD*)
• the power spectral density of the diameter in low [0.05-0.15 Hz] *(DLF*), high [0.15-0.45 Hz] (*DHF*), very high [0.45-1.5 Hz] (*DVHF*) frequencies ranges and the balance index (*DLF/DHF*)
• the normalized power spectral density of the diameter in low *(DLFn*) and high (*DHFn*) frequencies ranges.

Features in the frequency domain were obtained by computing the Welch’s periodogram of the detrended signal by using overlapped segments of 300 samples windowed with a Hamming window.

### C. Statistical Analysis

Two different comparisons were performed among the three different phases in the two emotional dimensions of valence and arousal. The aim was to assess how the body reacted according to different stimulation modalities such as only the view of images, only the listening to sounds or more complex stimuli given by the interaction of the two. For the arousal dimension, we compared features in each arousal session of one phase with the same arousal session of the other two phases. The same was done for the valence dimension: features computed during low and high valence of the same arousal session were compared among the three different phases. Specifically, Shapiro-Wilk test was performed for each feature values in low, high valence and in each entire arousal session to assess the normality of the data. Then, since always at least one variable was not normal distributed, the non parametric and pair-wise Friedman’s test was performed. In particular, if a comparison was significant, a multcompare comparison was then performed to assess pairwise differences. In all comparisons Tukey’s correction was applied.

## III. Results

Table 2 and Table 3 summarize all the relevant features found in the comparison among the three phases related to different stimulation modalities for the arousal and valence dimensions, respectively.

**TABLE 2:** Median and Ranges of all the Features Computed in the Arousal Sessions for the Three Stimuli. Statistically Significant Differences are Shown in Bold and the Last Column Specifies Between Which Phases These Differences are Observed (1: IAPS-Only, 2: IADS-Only, 3: IAPS+IADS)

AROUSAL	IAPS-only				IADS-only				IAPS+IADS				*
	A1	A2	A3	A4	A1	A2	A3	A4	A1	A2	A3	A4	
ECG													
$\mu $RR [ms]	812 (131)	817 (154)	847 (189)	835 (128)	862 (198)	847(200)	858 (187)	856 (154)	837 (146)	846 (130)	848 (162)	841 (139)	–
RR LFn [ms^2^/Hz]	0.78(0.29)	0.69(0.26)	0.74(0.26)	0.71(0.38)	0.64(0.31)	0.74(0.23)	0.73(0.23)	0.65(0.34)	0.74(0.20)	0.68(0.20)	0.69(0.24)	0.71(0.20)	2vs.3(A4)
RR HFn [ms^2^/Hz]	0.22(0.29)	0.31(0.26)	0.26(0.26)	0.28(0.38)	0.36(0.31)	0.26(0.23)	0.27(0.23)	0.35(0.34)	026(0.20)	0.32(0.21)	0.31(0.24)	0.29(0.20)	2vs.3(A4)
LF/HF	3.81(4.49)	2.49(1.99)	2.65(4.17)	3.22(4.49)	2.02(4.01)	3(3.1)	2.99(3.993)	2.03(2.76)	3.02(3.89)	2.21(1.98)	2.80(3.59)	2.78(2.93)	2vs.1,3(A4)
BVP													
VP [a.u.]	6.87(4.35)	5.93(4.87)	6.72(4.97)	5.38(4.44)	5.02(3.96)	4.78(5.43)	4.62(4.52)	5.64(3.99)	6.19(3.66)	5.66(4.39)	5.89(4.27)	5.33(3.48)	–
PAT [ms]	292(43)	292(43)	299(44)	300(42)	296(41)	295(41)	296(46)	297(46)	296(46)	296(44)	296(45)	298(45)	2vs.1(A3)/2vs.3(A2,A4)
GSR
GSR Amp peaks [nS]	0(6.9)	0(12)	0(24)	1.4(22)	20(49)	5.7(29)	4.2(44)	3.2(33)	2.7(35)	0(60)	1.1(10)	2.5(53)	1vs.2(A1,A3)
GSR n peaks	0(1.37)	0(2)	0.5(3.12)	1(2.5)	0.5(3.25)	1(3)	0.5(3.12)	0.5(2.62)	0.5(3)	0(3)	0.5(2.75)	0.5(3.25)	2vs.1,3(A1)
AV env [nS]	3.4(10)	2.4(16)	5(23)	4.4(30)	3.8(43)	5.6(32)	3.3(37)	5.3(27)	4.4(22)	3.1(47)	7.2(20)	17(63)	1vs.2(A1,A2)
PUPIL													
Std diameter [mm]	0.22 (0.11)	0.24 (0.10)	0.26 (0.12)	0.28 (0.10)	0.22 (0.13)	0.24 (0.11)	0.23 (0.10)	0.26 (0.13)	0.23 (0.11)	0.26 (0.10)	0.25 (0.11)	0.26 (0.14)	2vs.1,3(A3)
DVHF [mm^2^/Hz]	0.26 (0.27)	0.31 (0.31)	0.31 (0.35)	0.36 (0.37)	0.26 (0.49)	0.38 (0.35)	0.41 (0.37)	0.31 (0.34)	0.32 (0.39)	0.28 (0.37)	0.40 (0.46)	0.41 (0.42)	1vs.3(A4)
EEG													
$\beta /\theta $ frontal	0.18 (0.10)	0.18 (0.15)	0.18 (0.20)	0.24 (0.11)	0.42 (0.17)	0.38 (0.21)	0.47 (0.16)	0.26 (0.08)	0.16 (0.11)	0.18 (0.13)	0.17 (0.13)	0.19 (0.10)	2vs.1,3(A1,A2,A3)
$\beta /\theta $ parietal	0.30 (0.20)	0.33 (0.23)	0.29 (0.20)	0.35 (0.04)	0.52 (0.20)	0.47 (0.18)	0.46 (0.22)	0.33 (0.13)	0.30 (0.11)	0.32 (0.08)	0.31 (0.10)	0.35 (0.14)	2vs.1,3(A1,A2,A3)

**TABLE 3:** Median and Ranges of all the Features Computed in Low (L) and high (H) Valence Sessions for the Three Stimuli. For the Sake of Clarity, Only Features That Showed Statistically Significant Differences are Shown. Statistically Significant Differences are Shown in Bold and the Last Column Specifies Between Which Phases These Differences are Observed (1: IAPS-Only, 2: IADS-Only, 3: IAPS+IADS)

VALENCE	IAPS-only				IADS-only				IAPS+IADS				*
	A1	A2	A3	A4	A1	A2	A3	A4	A1	A2	A3	A4
BVP													
L: VP [a.u.]	6.92 (4.76)	7.11 (4.50)	6.79 (5.20)	5.31 (3.95)	4.89 (4.02)	4.38 (5.77)	4.61 (4.80)	5.54 (4.12)	6.60 (3.76)	6.45 (4.42)	6.58 (4.81)	6.11 (3.89)	2vs.3(A1)
H: PAT [ms]	293 (40)	293 (39)	300 (45)	299 (42)	296 (41)	298 (40)	297 (47)	296 (45)	297 (43)	295 (46)	296 (43)	298 (47)	1vs2(A3)/2vs.3(A2,A4)
GSR													
H: GSR Amp peaks [nS]	0 (1)	0 (8)	0 (20)	0 (22)	27 (54)	4 (34)	0 (45)	0 (36)	0 (14)	0 (52)	0 (14)	5 (61)	1vs.2(A1,A2)
H: GSR n peaks	0 (1.25)	0 (1.25)	0 (2.25)	1 (2)	1 (2.50)	1 (3)	0 (3.25)	0 (3)	0 (2.5)	0 (3)	0 (2)	1 (3.25)	1vs.2(A1),2vs3(A3)
H: AVenv [nS]	3 (9)	2 (13)	3 (22)	6 (19)	6 (36)	4 (37)	3 (44)	4 (24)	5 (24)	3 (46)	7 (26)	22 (68)	1vs.2(A1)
H: GSR rise Time [ms]	0 (271)	0 (644)	0 (944)	375 (881)	850 (1125)	875 (1311)	0 (1039)	0 (1063)	0 (750)	0 (1025)	0 (960)	750 (961)	1vs.2(A1)
PUPIL													
L: DLF [mm^2^/Hz]	0.28 (0.23)	0.33 (0.51)	0.38 (0.63)	0.45 (0.57)	0.32 (0.42)	0.42 (0.43)	0.54 (0.54)	0.27 (0.73)	0.26 (0.67)	0.28 (0.48)	0.30 (0.62)	0.30 (0.50)	2vs1,3(A4)
L: SDD [mm]	0.22 (0.11)	0.24 (0.10)	0.26 (0.12)	0.28 (0.10)	0.23 (0.13)	0.24 (0.10)	0.23 (0.10)	0.26 (0.13)	0.23 (0.11)	0.26 (0.10)	0.25 (0.11)	0.26 (0.14)	2vs1,3(A4)
H: DLF [mm^2^/Hz]	0.28 (0.23)	0.33 (0.51)	0.38 (0.63)	0.45 (0.57)	0.32 (0.42)	0.42 (0.43)	0.54 (0.54)	0.27 (0.73)	0.26 (0.67)	0.28 (0.48)	0.30 (0.62)	0.30 (0.50)	1vs.3(A3)
H: DHF [mm^2^/Hz]	0.34 (0.22)	0.33 (0.32)	0.41 (0.56)	0.44 (0.44)	0.35 (0.49)	0.44 (0.35)	0.34 (0.42)	0.26 (0.43)	0.32 (0.44)	0.39 (0.36)	0.40 (0.25)	0.47 (0.42)	1vs.2(A3)
EEG													
H: $\beta /\theta $ frontal	0.20 (0.14)	0.21 (0.14)	0.16 (0.19)	0.21 (0.11)	0.35 (0.17)	0.36 (0.23)	0.34 (0.21)	0.19 (0.15)	0.16 (0.11)	0.19 (0.10)	0.17 (0.17)	0.18 (0.05)	2vs.1,3(A1,A2,A3)
H: $\beta /\theta $ parietal	0.30 (0.24)	0.33 (0.13)	0.29 (0.37)	0.32 (0.10)	0.48 (0.16)	0.43 (0.20)	0.50 (0.21)	0.32 (0.13)	0.27 (0.10)	0.32 (0.09)	0.33 (0.09)	0.32 (0.08)	2vs.3(A1,A2)/2vs.1,3(A3)
L: $\beta /\theta $ F	0.19 (0.23)	0.17 (0.17)	0.17 (0.23)	0.26 (0.14)	0.41 (0.22)	0.35 (0.20)	0.42 (0.35)	0.27 (0.24)	0.20 (0.12)	0.16 (0.14)	0.15 (0.06)	0.18 (0.11)	2vs.1,3(A1,A2)
L: $\beta /\theta $ P	0.32 (0.16)	0.33 (0.18)	0.31 (0.21)	0.34 (0.05)	0.53 (0.21)	0.42 (0.25)	0.40 (0.33)	0.36 (0.16)	0.33 (0.16)	0.31 (0.08)	0.28 (0.07)	0.36 (0.08)	2vs.1,3 (A1)/2vs.3(A2,A3)

In the followings, results are shown divided by signal and for emotional dimension, in terms of arousal and valence. Figure 4 displays four features related to GSR, ECG, BVP, and PUPIL signals, which exhibited greater significance in delineating differences during the same arousal session across the three types of stimulation. These distinctions, as observed in the image, are presented in more detail below within the context of their respective signals.

**FIGURE 3.**
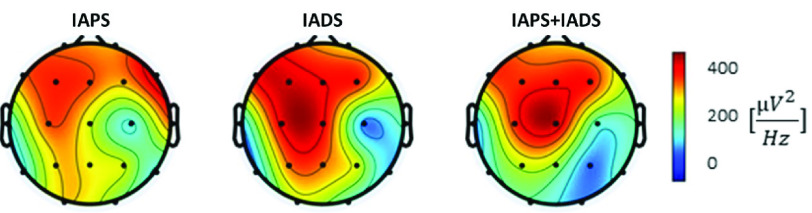
Activation on the scalp in terms of Power Spectral Density in $\delta $ band for each type of stimulation mode averaged among subjects.

**FIGURE 4.**
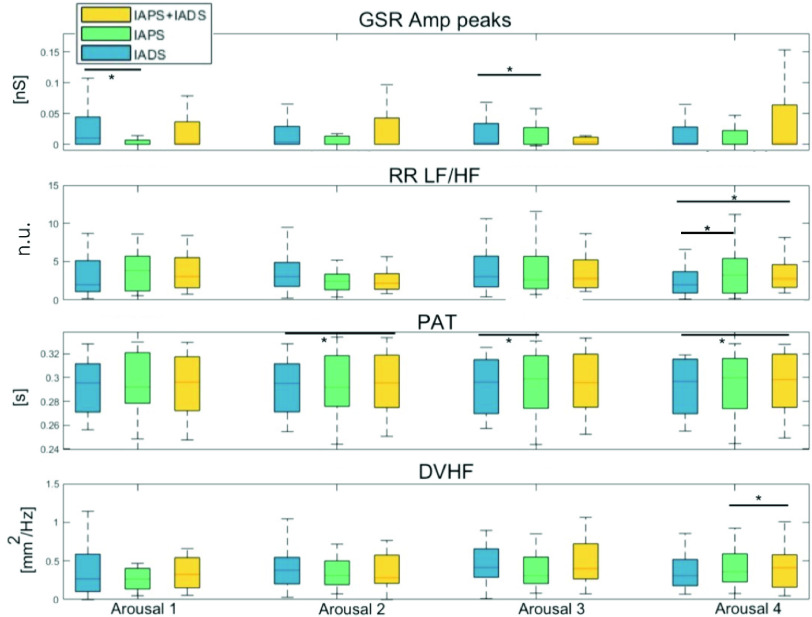
Distributions of the features that showed statistically significant differences in arousal among the three phases. From top to buttom: GSR (GSR Amp peaks), ECG (RR LF/HF), BVP (PAT) and PUPIL (DVHF). Statistically significant differences are marked with *.

*GSR Arousal*: From Table 2 it is possible to notice that *GSR Amp peaks* are significantly higher in IADS-only in A1 and A3 with respect to IAPS-only and the same behavior is detectable for *AVenv* which is always significantly greater in IADS-only than in IAPS-only in A1 and A2. *GSR n peaks*, moreover, are significantly higher not only in IADS-only but also in IAPS+IADS with respect to IAPS-only during A1. A general lower activity is observed for GSR in the phase involving only the sight of pictures.

*GSR Valence:* Table 3 shows how significantly higher *GSR Amp peaks*, *GSR n peaks*, *AVenv* and *AVGSR rise time* are observed during high valence stimuli of IADS-only with respect to IAPS-only in A1. *GSR Amp peaks* are higher for high valence stimuli also in A2 during IADS-only with respect to IAPS-only. Overall, high valence stimuli, linked to positive emotions, seem to elicit a higher GSR physiological response.

*ECG Arousal*: Table 2 shows how $\mu $RR has a general higher trend in IADS-only with respect to the other two phases. In all arousal sessions, median values are higher in IADS-only. We can state that a deceleration of the heartbeat is therefore observed when subjects listen to sounds alone. A lower sympathetic activity for IADS-only is also visible if we look at the time-varying spectral indices. *RR LFn* shows significantly lower values during IADS-only with respect to IAPS+IADS in A4. According to the same two phases and the same arousal session a significant opposite behavior is observed for *RR HFn*. Also *RR LF/HF* is significantly lower in IADS-only with respect to the other two phases in A4 (see Figure 4).

No statistically significant difference is observed in the valence dimension.

*BVP Arousal*: from the ECG-BVP interaction the computed PAT is significantly lower in IADS-only with respect to IAPS-only in A3. The same significant behavior is observed between IADS-only and IAPS+IADS in A2 and A4 (see Figure 4 and Table 2). No statistically significant difference was found for VP as shown in Table 2. However, this feature shows a general trend of low values in IADS-only with respect to the other two phases. Despite deceleration of the heartbeat, the blood propagation time from the heart to the periphery narrows and the blood volume decreases during the beats related to IADS-only.

*BVP Valence*: From Table 3 it is possible to notice significantly lower *PAT* values in IADS-only during high valence stimuli in A2 and A4 with respect to IAPS+IADS and in A3 with respect to IAPS-only. Significantly lower *VP* values are observed in IADS-only during high valence stimuli in A1 with respect to IAPS+IADS. In general we observe lower values during IADS-only in all arousal sessions with respect to the other two phases and this behavior seem to be attributed to high valence stimulation.

*EEG arousal*: The average activation during all arousal sessions on the scalp in $\delta $ has been reported in Figure 3. We can qualitatively notice that for all types of stimulation the most activated areas are the frontal and central, with a more pronounced activation in IADS-only.

**FIGURE 5.**
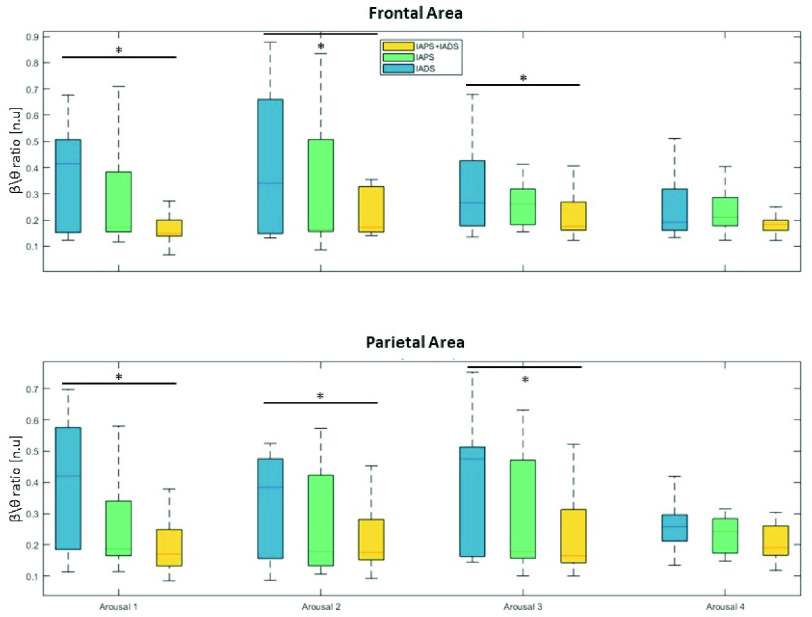
Distributions of the Attention index $\beta /\theta $ that showed statistically significant differences among phases in parietal and frontal areas. Statistically significant differences are marked with *.

The power spectral density computed in $\delta $, however, showed no statistically significant differences between IAPS-only, IADS-only and IAPS+IADS. Nevertheless, the main significant differences have been highlighted for the attention features extracted (see Table 2). In particular, it is worth to note that IADS-only resulted always in higher attention levels (in both frontal and parietal areas), beside the forth arousal level where no difference can be assessed. In this regard, Figure 5 displays distributions of the Attention index $\beta /\theta $ in the frontal and parietal lobes for the three types of stimulation. It is evident that in the IADS-only phase, this index tends to be significantly higher compared to the IAPS+IADS phase, with a clear upward trend also when compared to the IAPS-only phase.

*EEG valence*: Low valence stimulation resulted in different responses depending on the area analyzed (see Table 3). For the frontal area IADS-only stimulation resulted in significantly higher attention levels during the first and second arousal sessions, while in the parietal areas it was higher than the other two types of stimulations only in the first arousal and higher of IADS+IAPS only in the second and third arousal sessions.

Similar results have been obtained during high valence stimuli. In the frontal area IADS-only is significantly higher than the other two phases during A1, A2 and A3, while for the parietal in A1 it showed significantly higher attention levels only if compared to IAPS+IADS stimulation and in A2 and A3 the attention level in IADS-only is significantly higher than the other two phases.

*PUPIL Arousal*: *AVD* is significantly lower in each arousal session during IADS-only with respect to the other two phases. No statistically significant difference is observed between IAPS-only and IAPS+IADS in any of the arousal sessions. Diameter values are not reported in Table 2 since the comparison with IADS-only was not fair since during listening to sounds only subjects were looking at a gray screen so as expected the pupillary excursion is minor in this phase than in the other two. SSD is significantly lower during IADS-only than in the other two phases in A3. With regard to frequency features, *DVHF* is significantly higher in IAPS+IADS with respect to IAPS-only in A4 (Figure 4). In particular this feature, not very much investigated in the literature, seems to have a general increase at increasing arousal.

**FIGURE 6.**
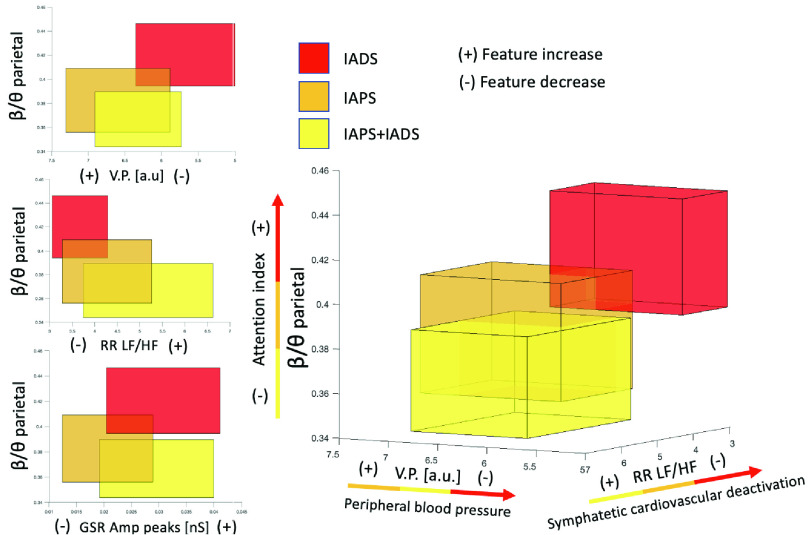
Exemplary 2D and 3D boxplots of features from ECG, GSR, BVP, and EEG that show differences in physiological responses between different stimuli.

*PUPIL Valence*: During high valence stimulation Table 3 shows a significantly higher *DHF* during IAPS-only with respect to IADS-only in A3. Always in A3, DLF is significantly higher during IAPS-only with respect IAPS+IADS. For *DLFn*, *DHFn* and *DLF/DHF*, though, even if no statistically significant difference is observed, we observe an agreement with higher excitation during IADS-only in A3: higher values for *DLFn* and *DLF/DHF* and lower values for *DHFn*. On the other hand, for low valence stimuli we can observe significantly higher DLF during IAPS-only with respect to both other phases in A4. Of note, the same behavior is not observed for *DLFn* and *DLF/DHF* in A4. Indeed, even if no statistically significant difference is found for *DLFn* and *DLF/DHF*, both features show an increase during IADS-only with respect to the other two phases, whereas the opposite trend is observed in both *DHF* and *DHFn*, i.e. lower values during IADS-only. Finally, pupillary diameter s.d. is found to be significantly higher during IAPS-only with respect to the other two phases in A4. Figure 6 shows the main results obtained in the study. In particular, both 2D and 3D boxplots were created by joining all feature values of the four arousal sessions of each phase (mean ± standard error). Figure 6 displays features that best explain the physiological behavior linked to the different stimulations. The attention index increases when transitioning from IAPS+IADS to IAPS-only and further attains higher values during the IADS-only phase, which is entirely distinct from the IAPS+IADS phase where the stimulus becomes more complex by involving both visual and auditory senses. It is also important to observe that from a cardiovascular perspective, at the peripheral vessel level, the IADS-only phase is associated with lower values of VP, which are linked to an increase in blood pressure [28]. This is, however, connected to a more centrally located lower RR LF/HF index derived from the ECG, indicating reduced sympathetic activity. From the perspective of eccrine glands, lower activity is observed during the IAPS-only phase.

## IV. Discussion

Overall, our results from the analysis of physiological signals such as GSR, ECG, BVP, PUPIL and EEG, demonstrate that sounds tend to elicit higher emotional responses than visual stimuli, either alone or in combination with sounds. A unique aspect is the use of the Point process framework to analyze ECG data, which is rarely used for HRV analysis in this context. Unlike traditional methods used in shorter time windows, this framework adheres to established guidelines for HRV analysis, allowing real-time extraction of HRV indices [29].

By looking at the results from GSR, we can speculate that higher sympathetic activity is elicited when listening to sounds compared to watching images. Looking at the arousal dimension, indeed, *GSR Amp peaks* and *AVenv* (which are related to the spikes of the eccrine glands and thus linked to sympathetic activation), result to be higher during IADS-only with respect to IAPS-only in two arousal sessions. Moreover, *GSR n peaks* results higher in IADS-only and IAPS+IADS with respect to IAPS-only in the first arousal session. By looking at the valence dimension, it seems that this higher excitation during the listening to sounds belongs to high valence stimuli, i.e., stimuli that carry a positive emotional content. For what concerns the ECG signal, it shows an opposite behavior with respect to GSR. Even if the average modeled RR series did not show any significant differences among the three stimulation modalities in any of the arousal sessions, there is an evident heartbeat deceleration during IADS-only. This behaviour is reflected also in the spectral indices: the normalized power spectral density in the low- and in the high-frequency ranges are significantly lower and higher, respectively, during IADS-only with respect to IAPS+IADS. Also the ratio between *RR LF/HF*, which is more related to the sympathetic branch of the autonomic nervous system is significantly higher in IAPS-only and IAPS+IADS with respect to IADS-only in the most arousing session A4. Conversely, by looking at *VP* from the BVP signal and from *PAT* derived from both ECG and BVP, is evident how the cardiovascular system compensates this heartbeat deceleration by increasing the blood pressure. By looking at Table 2, indeed, we can see how VP has lower values during IADS-only with respect to the other two phases. The amplitude modulation of the BVP signal represents, indeed, the volume of blood on the periphery and lower amplitudes are therefore linked to a greater peripheral blood pressure, which is associated with vasoconstriction. This effect is especially visible during low valence stimuli linked to negative emotions. By looking at Table 2 and Table 3 we can see how this compensation is reflected also in *PAT* which has lower values, especially at high valence. We can state that there is therefore an acceleration of the pressure wave since less time is spent by the blood to reach the periphery from the heart. By looking at the literature, this behavior could be referred to the attention theory. The Lacey’s theory of attention, indeed, has proved that the response of an organism to a task which involves attention is linked to a heart beat deceleration often accompanied by an increased GSR [30]. According to Laceys’ theory [31], this deceleration occurs due to the acceptance of environmental stimuli which require attention such as, for instance, perception of a visual or auditory stimulus. In terms of auditory involvement, emotional sounds are processed more rapidly and directly than images, which involve more complex visual processing. This quicker engagement of the auditory system may result in heightened sympathetic and attentive activation [32]. Although few studies directly compare visual and auditory emotional stimuli, the literature offers diverse insights. In [33], findings suggest that audio and visual stimuli trigger comparable stress responses. Conversely, [34] emphasizes asymmetrical emotional responses to pictures and sounds, where pupillary dilation is more associated with arousal in images and valence in sounds. Moreover, one of the few study on emotion elicitation which makes use of IAPS and IADS [16] found that auditory stimuli led to a higher speed of heart rate deceleration in comparison to visual stimuli as in our case. According also to the feedback provided by some of the subjects tested, sounds alone in the absence of a related image required them to focus and use their past memories and experiences to understand the meaning of the sound, thus eliciting stronger emotions. This assertion is confirmed by the response to emotional stimuli given by the central nervous system (i.e., EEG recordings). EEG did not show statistically significant differences between stimulation modalities in terms of brain activity in the brain areas and frequency bands usually related to emotion processing. Nevertheless, the activation along the scalp shows a better localized and higher response during IADS-only, suggesting that this type of stimulation may better evoke the expected emotion. Moreover, in most cases, it has been found a higher attention level of the subjects when stimulated with IADS-only. Regarding the pupillary signal, results are more difficult to compare and interpret since subjects were looking at a grey screen during IADS-only. Of note, the pupillary diameter is lower in all the arousal sessions for IADS-only with respect to the other two phases. The standard deviation of the diameter is lower during IADS-only as well. In general, features linked to pupillary amplitude seem to be not very representative since no statistically significant difference is observed between IAPS-only and IAPS+IADS, so sounds do not seem to influence the pupil diameter. About the frequency content of the pupillary signal, it seems that higher power spectral density is observed during IAPS-only both in low and high frequency ranges with respect to the other two phases in some arousal sessions. However, by looking at the normalized power spectral densities, even if not significantly, it seems that IADS-only is the one associated with greater values of *DLFn* and lower values of *DHFn* for the most arousing sessions (e.g. A3 and A4), thus emphasizing a greater sympathetic control also on the pupil when listening to sounds only. Moreover, statistically significant differences are observed in the very high frequency range in which higher differences are observed during IAPS+IADS compared to IAPS-only. Since this frequency range may not be associated with the autonomic nervous system, this point should be investigated further. Figure 6 provides an intuitive graphical summary of the main results obtained. Specifically, IADS-only is associated with sympathetic cardiovascular deactivation, which is compensated by an increase of the peripheral blood pressure. Moreover, increased GSR peak amplitudes in IADS-only are observed, especially with respect to IAPS-only. This behavior is explained by Lacey’s theory of attention and reflected at the central level by looking at the EEG where the attention index turned out to be greater when listening to sounds. It therefore seems that when listening to sounds subjects pay more attention and are more stimulated. These results are encouraging and support the use of a multimodal signal processing approach to investigate physiological responses. While EEG predictably offered the most significant results, less invasive signals such as GSR, BVP, and ECG still provided valuable insights into physiological patterns. Monitoring central and peripheral signals improved our grasp of physiology, aligning with Lacey’s theory that ECG and GSR alone can capture attention changes without more cumbersome EEG procedures. In any case, the main focus of our study was about understanding the effects and impact of different stimulation modalities, rather than investigate signals reliability.

From a clinical perspective, our finding that emotional sounds elicit stronger physiological responses than visual and audio-visual stimuli holds promise for clinical applications. For example, proper sound protocols could enhance mood disorder treatments that are often only based on visual clues [35], potentially leading to improved emotional regulation with a specific focus on a personalized set of sound stimuli. An approach based on personalized sound stimulation can also be particularly relevant in neurofeedback therapy, especially for conditions like Attention Deficit Hyperactivity Disorder, where attention and self-control are of major concerns [36]. Moreover, individuals with disabilities, including those on the autism spectrum, may benefit from sound-based therapies, as suggested by studies combining music activities with sound therapy [37]. The implications of these findings extend beyond clinical practice. For example, the use of sound stimuli could bring new opportunities in neuromarketing, where the emotional impact of sounds could be leveraged to design personalized, more effective marketing strategies.

## V. Conclusion

We here present a new acquisition protocol in order to compare three different stimulation modalities to convey emotional content through images, sounds and a combination of them. In particular, we focus on the acquisition of ECG, BVP, GSR, PUPIL, and EEG to understand which stimulation is the most effective. By extracting features linked to both the autonomic and central nervous systems we found how sounds are able to stimulate more at a physiological level. From the GSR signal it is evident how the phase of only sounds is more linked to a sympathetic activation than the other two phases. On the other hand, at the cardiovascular level there is a deceleration of the heartbeat, which is accompanied by a compensation given by a minor blood volume amplitude at the peripheral level, and a greater speed of blood from the heart to the periphery. At the central level, the relevant result is that a greater attention index is observed while listening to sounds. We can conclude by saying that sounds seem to stimulate the subjects more, placing greater importance on this type of stimulation where very little has been investigated in the literature. Overall, we conclude that sounds convey emotional content and reveal to be very effective in eliciting specific emotions, thus confirming their potential effectiveness in different fields of psychophysiology. The main limitation of this study is that acquisitions were conducted in a laboratory, where subjects may be little induced to feel emotions being not in a natural environment. Another limitation of the study is the absence of a detailed assessment of the participants’ mental health and emotional state, which could have allowed to identify the potential presence of response outliers and support a more precise interpretation of individual results. Future research is needed to further validate the findings of this study through an in-depth analysis of possible confounding variables in a larger sample of participants. Moreover, it will be important to identify strategies for more personalized stimulation, for example by optimizing sound stimuli on an individualized basis and by addressing the possible influence of the individual emotional state. Finally, it will be important to create more immersive protocols, such as in virtual reality environments, that can provide an even more effective and emotion-targeting stimulation.
